# Predictors of pleural complications in trauma patients undergoing tube thoracostomy: A prospective observational study

**DOI:** 10.1590/0100-6991e-20223300-en

**Published:** 2022-08-17

**Authors:** ISIS KOBASHIGAWA DO NASCIMENTO, HELENA MACHADO MORAD, JACQUELINE ARANTES GIANNINNI PERLINGEIRO, JOSÉ GUSTAVO PARREIRA, TCBC-SP, JOSE CESAR ASSEF

**Affiliations:** 1 - Faculdade de Ciências Médicas da Santa Casa de São Paulo, Cirurgia - São Paulo - SP - Brasil; 2 - Irmandade da Santa Casa de Misericórdia de São Paulo, Serviço de Emergência - São Paulo - SP - Brasil

**Keywords:** Thoracic Injuries, Thoracostomy, Hemothorax, Pneumothorax, Postoperative Complications, Traumatismos Torácicos, Toracostomia, Hemotórax, Estudo Observacional, Complicações Pós-operatórias

## Abstract

**Objective:**

to identify variables related to pleural complications in patients undergoing tube thoracostomies due to traumatic injuries.

**Method:**

we conducted a prospective observational study from May/2019 to January/2021 including adult trauma patients submitted to tube thoracostomies after hospital admission. Patients undergoing thoracotomies as the initial treatment were not included. We excluded patients with suspected and confirmed COVID-19 diagnosis during the hospitalization. Pleural complications were defined as clotted hemothorax, residual pneumothorax and empyema. Students t, Mann Whitneys, Chi square and Fishers exact test were used to compare variables between groups. We considered p<0.05 as significant.

**Results:**

we analyzed 68 patients. The mean age was 36.0 + 12.6 years and 91.2% were male. The mean RTS and ISS were, respectively, 7.0 ± 1.6 and 15.9 ± 7.6. The most frequent trauma mechanism was stab wounds in 50.0%, followed by blunt trauma in 38.2%. The severity of thoracic injuries was stratified (AIS) as 2 (4.4%), 3 (80.9%), 4 (13.2%), e 5 (1.5%). Pleural complications happened in 14 (20.5%) patients, being clotted / residual hemothorax (11.8%), residual pneumothorax (4.4%), empyema (2.9%) and miscellaneous (1.4%). These patients were treated by thoracoscopy (5), thoracotomy (3), chest re-drainage (3) and clinical measures alone (3). There was a significant association between pleural complications with the time of permanence (p<0,001) and the necessity of relocation (p<0,001) of the drain.

**Conclusion:**

the predictors of pleural complications in this series were time of permanence and the necessity of relocation of the drain.

## RESUMO

**Objetivo:**

identificar as variáveis relacionadas às complicações pleurais em vítimas de trauma submetidas a drenagem torácica.

**Método:**

estudo observacional prospectivo entre maio/2019 e janeiro/2021, incluindo adultos submetidos a drenagem torácica após a admissão hospitalar para tratamento de lesões traumáticas. Pacientes que desenvolveram COVID19 foram excluídos. As complicações pleurais foram caracterizadas como: pneumotórax residual, hemotórax residual / coagulado e empiema pleural. Comparamos as variáveis coletadas entre os grupos de pacientes com e sem estas complicações. Os testes t de Student, Mann Whitney, Chi quadrado e Fisher foram utilizados para análise estatística. Foram considerados significativos valores de p<0,05.

**Resultados:**

analisamos 68 casos, com média etária de 36,0 + 12,6 anos, de RTS de 7,0 ± 1,6 e ISS de 15,9 ± 7,6, sendo 62 (91,2%) do sexo masculino. Trinta e quatro pacientes (50,0%) foram vítimas de ferimento por arma branca. O AIS das lesões torácicas foi classificado como 2 (4,4%), 3 (80,9%), 4 (13,2%), e 5 (1,5%). As complicações pleurais ocorreram em 14 casos (20,5%), sendo hemotórax residual / coagulado em 8 casos (11,8%), pneumotórax residual em 3 (4,4%), o empiema pleural em 2 (2,9%) e associações em 1 (1,4%). Estas complicações foram tratadas por videotoracoscopia (5 casos), toracotomia (3), redrenagem de tórax (3) e medidas clínicas apenas (3). A redrenagem de tórax (p<0,001) e o tempo de permanência do dreno (p<0,001) tiveram relação significativa com a presença de complicações pleurais.

**Conclusão:**

a redrenagem do tórax e o maior tempo de permanência do dreno foram associados às complicações pleurais.

## INTRODUCTION

Trauma is a frequent cause of death, sequelae, hospitalizations, and expenses in Brazil^1^ ^,^ ^2^. The thorax is one of the most affected body segments, with risk of compromising vital organs such as the heart, great vessels, and lungs^3^. The most common injuries are hemothorax, pneumothorax, and rib fractures^4^ ^,^ ^5^.

Chest drainage is the most frequent surgical procedure in the treatment of chest injuries, being performed in 40% to 66% of cases^4^ ^-^ ^6^. It is considered technically simple and capable of saving lives. Complications occur in 5% to 35%, involving technical failures, malposition of the chest tube, iatrogenic injuries, retained or clotted hemothorax, non-expansion of the lung (residual pneumothorax), and pleural empyema^7^ ^-^ ^10^. Some factors are associated with these failures, such as the surgeon’s inexperience, errors in the identification of anatomical structures, the drainage performed in the admission room, the type of drain used, emergency, and the stress of the surgical team^8^ ^,^ ^9^ ^,^ ^11^.

Pleural complications that can result from chest drainage increase the length of hospital stay, costs, the frequency of unscheduled reoperations, and deaths. The application of specific care protocols reduces the frequency of these adverse events^9^ ^,^ ^12^ ^,^ ^13^. However, many resources are not available to all patients in our country. The identification of variables related to pleural complications allows us to select patients at higher risk early, directing efforts to reduce adverse effects.

The aim of this study is to identify variables related to pleural complications in trauma victims undergoing chest drainage.

## METHODS

This study was approved by the Ethics in Research Committee of our institution (Opinion Number: 3.304.287 / CAAE: 11867919.1.0000.5479).

We performed a prospective, observational study between May 2019 and January 2021. We included all adult (age >17 years) victims of trauma (blunt or penetrating) admitted to the emergency room directly from the scene and undergoing chest drainage in the first six hours for the treatment of hemothorax / pneumothorax resulting from the event. We did not include those who did not sign the informed consent form, patients drained in the prehospital setting, those with initial treatment by thoracotomy or sternotomy, those who had chest drainage because of iatrogenic injuries (eg. central catheter insertion), cases initially treated non-operatively, and those with coagulopathy and use of anticoagulants. We excluded those who intentionally removed the chest drain due to lack of cooperation with the treatment and those who were diagnosed with COVID-19 during hospitalization.

In our institution, water-seal chest drainage is indicated for the treatment of traumatic hemothorax and pneumothorax, diagnosed clinically or by imaging tests. There are exceptions in trauma victims with occult pneumothoraces (not identified on radiography, but only on chest tomography), small-volume pneumothorax in asymptomatic patients, and small-volume hemothorax (<2.5cm in length, accumulated in the posterior region at computed tomography). The surgical procedure is performed under local anesthesia according to the technique standardized by the Advanced Trauma Life Support (ATLS) 10^th^ edition^14^, followed by an anteroposterior chest X-ray to assess drain location. If the drain is not properly functioning (eg, without oscillation of the column in the water seal), with holes in the subcutaneous tissue, or in an atypical location, the drain is promptly relocated. The chest tube is removed after lung expansion (clinical and radiological) and a output of less than 150ml/24h. After removal, the patient undergoes a new chest X-ray in 12 hours.

We collected data on sex, age, trauma mechanism (blunt/penetrating, gunshot wound, stab wound, or other), clinical variables at emergency room admission (vital signs, Glasgow Coma Scale)^15^, Revised Trauma Score (RTS)^16^, identified traumatic injuries and their stratification by the Abbreviated Injury Scale (AIS)^17^ and the Injury Severity Score (ISS)^18^, chest drainage site (emergency room or operating room), surgical procedure variables (asepsis and dressing, use of systemic sedation, need for orotracheal intubation), procedure technique (intercostal space of insertion, gauge and positioning of the drain), surgeon experience (resident or staff), chest X-ray performed before the procedure, need for chest redrainage, pre-existing pulmonary comorbidities, technical complications (visceral injuries, subcutaneous orifices, fixation failure), general complications, and deaths.

Pleural complications were defined as:

- Residual pneumothorax: no lung expansion within 48 hours of drainage or identified by chest X-ray after removal of the chest tube;
- Residual or clotted hemothorax: presence of hemothorax after 48 hours of chest drainage, confirmed by chest tomography;
- Complicated pleural effusion or empyema: presence of complicated pleural effusion (Light’s criteria) or even empyema by clinical and laboratory diagnosis (thoracentesis or chest drainage), without a compatible thoracic or abdominal injury (eg esophageal injury); and
- Associations: cases in which there were associations of pneumothorax, residual hemothorax, and complicated pleural effusions.

At our institution, small-volume residual pneumothoraces are treated by optimizing analgesia and physical therapy, while medium- and large-volume pneumothoraces are submitted to chest redrainage. Retained or clotted hemothoraces of significant volume are treated by early thoracoscopy (ideally until the 4th or 5^th^ day), with a small thoracotomy if necessary for lavage. Complicated effusions and empyemas receive antibiotics and open chest drainage after the 10^th^ day and pleurostomy afterwards, depending on the evolution.

We divided patients into two groups:

- Group A: with pleural complications (sum of all types described)
- Group B: other patients

All cases were followed up by the authors during hospitalization. We also obtained data from the electronic medical records (MV^®^) and incorporated them into a Microsoft Excel spreadsheet for Windows, version 2010. At the end of the collection, the data were analyzed by the Biostatistics Service of Santa Casa of Sao Paulo School of Medical Sciences (FCMSCSP). We present data as mean ± standard deviation. In addition to descriptive statistics, we compared the variables of groups A and B to identify predictors of pleural complications. We used the chi-square or Fisher’s exact test to analyze categorical variables. We assessed continuous variables with the Student’s t or Mann Whitney tests, according to the normality of the sample. Values of p<0.05 were considered significant.

## RESULTS

The final sample consisted of 68 cases, with a mean age of 36.0 ± 12.6 years, RTS at admission of 7.0 ± 1.6, and of ISS of 15.9 ± 7.6, 62 patients (91.2%) being male (Table 1). Mean systolic blood pressure at admission was 115.4 ± 22.2 mmHg, respiratory rate 21.0 ± 6.1 breaths per minute, and Glasgow Coma Scale 13.0 ± 3.6. Thirty-four patients (50.0%) were victims of stab wounds, 26 (38.2%) of blunt trauma, and eight (11.8%) of gunshot wounds. Of the total, 52 (76.5%) were drained in the emergency room and 16 (23.5%) in the operating room. Of all the procedures, 50 (73.5%) were performed by the first-year general surgery resident (R1), 11 (16.2%) by the R2, five (7.4%) by the R3, one (1.5%) by the R4, and one (1.5%) by medical intern (under medical supervision). The mean drain length of stay was 6.3 ± 6.1 days.

**Table 1:** General sample data.

General sample data	Description
Male sex	62 (91.2 %)
Penetrating injury	42 (61.7 %)
Abdominal injury	26 (38.2 %)
Chest drainage in the emergency room	52 (76.4 %)
Sedation and OTI	31 (45.5 %)
Postoperative antibiotics	40 (58.8 %)
Performed by R1	50 (73.5 %)
Previous lung disease	8 (11.8 %)
Chest AIS = 3	55 (80.9 %)
Insertion of the drain in the 5th intercostal space	40 (58.8 %)
Chest X-ray before drainage	51 (76.1 %)
Thoracic redrainage	16 (23.5 %)
Pleural complications	14 (20.5 %)
Residual pneumothorax	3 (4.4 %)
Residual hemothorax	8 (11.8 %)
Empyema	2 (2.9 %)
Deaths	11 (16.1 %)

OTI: Orotracheal intubation; R1: first year general surgery resident; AIS: Abbreviated Injury Scale.

All procedures were performed under appropriate asepsis and attire. Of the total, 31 (45.6%) were sedated and intubated and 40 (58.8%) received postoperative antibiotics. The most frequent anatomical site of drain insertion was the fifth intercostal space (58.8%), followed by the sixth intercostal space (13.2%), fourth intercostal space (7.4%), and others (20.6%). Pre-drainage chest radiography was performed in 51 (76.1%) cases.

Of the 68 patients, eight (11.8%) had some previous lung disease. We observed associated lesions in the face (8.8%), head and neck (22.0%), external (11.7%), and skeleton/extremities (33.8%). Abdominal injuries were present in 38.2%. The AIS of chest injuries was classified as 2 (4.4%), 3 (80.9%), 4 (13.2%), and 5 (1.5%). Sixteen patients (23.5%) required chest redrainage. There were 11 (16.2%) deaths, none directly related to the chest injuries.

Pleural complications occurred in 14 cases (20.5%), with residual/coagulated hemothorax in eight (11.8%), residual pneumothorax in three (4.4%), pleural empyema in two (2.9%), and associations in one (1.4%). These complications were treated by thoracoscopy (five cases), thoracotomy (three cases), chest redrainage (three cases), and clinical measures only (three cases). None of these complications were directly related to any death.

Chest redrainage was required in 62.5% of the patients who developed pleural complications and in 7.8% of the others (p<0.001). The mean duration of the pleural drain was 12.1 ± 11.1 days in those with pleural complications and 4.7 ± 2.4 in the others (p<0.001) (Tables 2 and 3). The following variables did not display a statistical relationship with pleural complications: sex, previous lung disease, drain caliber, drain placement, use of antibiotics, surgeon’s experience, systemic sedation, orotracheal intubation, associated injuries (external integument, head and neck, face, abdomen, skeleton, and extremities), trauma mechanism, previous radiography, and drainage site (Tables 2 and 3).

**Table 2:** Comparison of qualitative variables between groups.

Variable	Group A n=14	Group B n=55	p-value*
Male sex	92.8%	90.7%	1,000
Penetrating mechanism	64.2%	38.8%	0.447
Abdominal injury	35.7%	37.0%	0.792
Emergency room drainage	78.5%	75.9%	1,000
Postoperative antibiotics	42.8%	62.9%	0.173
Surgeon 's experience (R1)	78.5%	72.2%	0.280
Sedation and OTI	57.1%	42.5%	0.330
Pre-drainage chest x-ray	85.7%	72.2%	0.490
Previous lung disease	14.2%	11.1%	0.762
chest re-drainage	62.5%	7.8%	<0.001
Death	14.2%	6.6%	1,000

Group A: patients with pleural complications; Group B: patients without pleural complications; *p-value derived from chi-square or Fisher tests; R1: first year general surgery resident; OTI: Orotracheal intubation.

**Table 3:** Comparison of quantitative variables between groups.

Variable	Group A	Group B	p value*
Age (years)	38.1 ± 9.6	35.5 ± 13.3	0.358
SBP (mmHg) admission	112.1 ± 15.5	116.3 ± 23.7	0.659
RF (ipm) admission	21.9 ±6.4	20.7 ± 6.1	0.460
GCS at admission	13.1 ±3.7	13.0 ±3.6	0.696
RTS	7.2 ±1.3	7.0 ± 1.6	0.810
ISS	13.9 ± 7.4	16.5 ± 7.6	0.199
Drainage time (days)	12.1 ± 11.1	4.7 ± 2.4	<0.001

Group A: patients with pleural complications; Group B: patients without pleural complications; *p value derived from Student’s t or Mann Whitney tests; RF: respiratory frequency; SBP: Systolic Blood Pressure; GCS: Glasgow Coma Scale; RTS: Revised Trauma Score; ISS: Injury Severity Score.

## DISCUSSION

The sample of this study is similar to those described in other series, with a predominance of young male adults, victims of penetrating injuries, and chest drainage performed in the trauma room^4^ ^,^ ^5^ ^,^ ^8^ ^,^ ^9^ ^,^ ^19^. Chest injuries classified as AIS=3 occurred in 80.9%. Most patients were hemodynamically stable at admission. In 76.1% of cases, chest radiography was performed before chest drainage.

The fact that 73.1% of the procedures were performed by first-year residents (R1 of General Surgery) may reinforce the mistaken idea that chest drainage is a procedure with few complications. The rate of pleural complications in our sample (20.5%) was significant and comparable to other series^8^ ^,^ ^9^ ^,^ ^19^. We did not observe serious iatrogenic injuries, such as intercostal vascular-nervous bundle lesion, lung parenchyma laceration, and diaphragmatic or intra-abdominal injury. However, in about 20% of the cases, the drain was inserted far from the location deemed correct by the ATLS 10^th^ Edition proposed technique^14^.

In our study, we did not find a significant relationship between the presence of pleural complications and factors that could be associated with them, such as age, surgeon experience, severity of chest injury (AIS), chest drainage site, drain caliber, pre-existing lung disease, among others in the data collected. Bell et al., in 2001, and Kong et al., in 2021, did not identify predictors of residual pneumothorax after drain removal on inspiration or expiration^20^ ^,^ ^21^. These authors also did not find another variable associated with this complication^20^. On the other hand, there are studies that have identified predictive factors for the development of thoracic post drainage empyema, such as the presence of retained hemothorax, pulmonary contusion, longer chest tube permanence, and need for exploratory laparotomy^22^. Menger et al., in 2012, identified the severity of chest injury classified by the AIS as the only independent factor related to post drainage complications^23^. There are studies that report a greater chance of late and after discharge rebleeding in approximately 1.4% of patients with chest injuries^24^ ^,^ ^25^.

The influence of chest drain caliber on the frequency of complications was also studied by other authors, who did not find differences between larger and smaller diameters^26^ ^,^ ^27^. In our study, we did not find a relationship between pleural complications and the size of the drains used. The new edition of the ATLS (10^th^ edition) already suggests smaller caliber drains for chest drainage^14^.

The only variables statistically associated with a higher frequency of pleural complications were the need for chest drainage (due to non-functioning drains) and the length of permanence of the drain. These variables could also be explained as the treatment of pleural complications and not just as their cause. However, it makes sense to say that chest redrainages due to malfunction increase the frequency of infection, as reported in the literature^22^.

Abreu et al., in 2015, evaluated the impact of applying a specialized care protocol for patients undergoing chest drainage in a public hospital, a reference for trauma, in the city of Belo Horizonte/Brazil^9^. These authors implemented items such as chest drainage in the operating room, antibiotic prophylaxis, and chest physiotherapy at least twice a day. There was a significant reduction in the incidence of retained hemothorax (31.3% vs. 6.5%), empyema (22.2% vs. 2.0%), pneumonia (11.1% vs. 0.0%), and drain permanence (4 days vs. 3 days). Molnar also emphasizes the importance of postoperative care in patients undergoing chest drainage: “The chest drainage procedure does not end at the last stich. The second half of the match is yet to come”^13^. Fitzgerald et al. draw attention to the importance of the operative technique in preventing post-drainage complications^28^. These authors emphasize the importance of asepsis, of avoiding the use of trocars, of performing a digital examination before introducing the drain into the pleural cavity, and of directing the drain in a posterior and cranial position.

Studies analyzed the use of ultrasound of the chest as part of the post-drainage care protocol with the aim of reducing the length of permanence of the drain^29^ ^,^ ^30^. Fonseca et al., in 2020, demonstrated a reduction in the length of stay of the chest drain from 4.9 to 2.5 days when ultrasound was used for decision making. Perhaps this could be an alternative, as pleural complications were associated precisely with longer permanence of the chest tube.

There are some limitations that we need to highlight in our study. The number of patients did not allow us to perform a more in-depth statistical analysis, such as logistic regression, to identify independent factors. It was a study developed in the middle of a pandemic and many patients diagnosed with COVID-19 were not included in the final sample. There were strict selection criteria to control for the many variables involved. Thus, we did not include patients treated directly by thoracotomy or sternotomy, as well as those who had initial nonoperative treatment and requiring late drainage.

Our results reinforce that the reduction of pleural complications also depends on measures taken during drainage. Staff must be trained to properly locate and secure the drain. The operative technique must be perfect. If the drain is not working, it must be repositioned right away. By avoiding late draining due to non-functioning drains, we reduced the length of stay, the chance of infection, and, consequently, pleural complications.
